# Intergenerational relationships after parental divorce: variations by levels of family solidarity

**DOI:** 10.1007/s10433-025-00849-x

**Published:** 2025-04-30

**Authors:** Zafer Buyukkececi

**Affiliations:** Max Planck Institute for Demographic Research, Rostock, Germany

**Keywords:** Parental divorce, Parent–child ties, Intergenerational solidarity, Recentered influence functions, Unconditional quantile regression

## Abstract

This study examines the association between gray parental divorce and family solidarity, defined as the quality and frequency of intergenerational relationships, including contact, emotional closeness, and functional support. The analysis focuses on how the effects of gray divorce vary across families with differing levels of closeness and interaction prior to the divorce, given that families with weaker bonds—such as infrequent contact or less emotional closeness—may face greater challenges in maintaining relationships after divorce compared to those with stronger ties. To test this, I use 13 waves of longitudinal data from pairfam and recentered influence function (RIF) regression that allows to examine how the effects of parental divorce differ across the entire distribution of solidarity, distinguishing between families with weak, moderate, and strong ties. Families with lower solidarity, particularly in terms of contact frequency and emotional closeness, are more likely to experience gray parental divorce. The negative effects of parental divorce on parent–adult child relationships are also more pronounced in these families and among fathers. Conversely, families with moderate or high levels of solidarity are more resilient to the negative impacts of divorce. Accordingly, the gap in intergenerational solidarity widens across families, with low-solidarity families being more likely to experience parental divorce later in life and exhibiting more negative outcomes compared to families with stronger ties. This growing differences across families also highlight the relevancy of targeted policy interventions aimed at supporting families, particularly those with weaker ties, and addressing the unique challenges faced by fathers after divorce.

**Supplementary Information:**

The online version contains supplementary material available at 10.1007/s10433-025-00849-x.

## Introduction

Intergenerational solidarity, referring to the degree of contact, closeness, support, and interdependence between generations, is an important aspect of family relationships and has been linked to numerous positive outcomes (Bengtson & Roberts 1991). Research has shown that strong parent–adult child relationships and family solidarity are linked to improved mental health and overall well-being, and reduced loneliness and depression for both generations (Bengtson 2001; Fingerman et al. 2020; Umberson et al. 2010). Over the life course, families provide emotional, instrumental, and social support, particularly during periods of stress or crisis (Minuchin 1985).

Yet, families are not static entities, and their structures and dynamics have changed significantly in recent decades. Complex family have emerged driven by rising rates of partnership dissolution, lone parenthood, re-partnering, and the formation of stepfamilies (Author, Raley & Sweeney 2020; Thomson 2014). Among these demographic shifts, gray divorce—divorce occurring after age 50—has become increasingly common in Western countries, including Germany (Bundesinstitut für Bevölkerungsforschung 2022).

Divorce is often a source of stress with implications for well-being (Leopold 2018) and other family subsystems, including parent–adult child relationships (Buyukkececi & Leopold 2024; Lin et al. 2022). Those experiencing gray divorce often find themselves living alone without the support of a spouse, which can increase feelings of isolation and vulnerability (Silverstein et al. 2006). These trends highlight the importance of adult children as sources of emotional and practical support, especially for fathers, who are more susceptible to the negative consequences of divorce, including health declines and diminished well-being (Shor et al. 2012).

Accordingly, understanding the consequences of gray parental divorce for parent–child relationships is increasingly important. Early studies (Angell 1936; Hill 1949) suggest that families with stronger ties are more adaptable and better equipped to cope with major transitions and crises, such as parental divorce. Family support can help buffer the adverse effects of stressful life events on individuals and families. In contrast, individuals with weaker family ties and limited social support may face more severe consequences, as stressful events can enhance existing vulnerabilities (Cohen & Hoberman 1983; Cohen & Wills 1985).

Despite the recognized importance of this topic, little attention has been given to how the effects of parental divorce vary across families with differing levels of solidarity. Families with weaker ties may not only be more likely to experience parental divorce but also more severely affected by it, which could, in turn, increase disparities across families. Accordingly, parental divorce may deepen inequalities, disproportionately impacting less cohesive families and increasing their vulnerability to future challenges.

This study addresses gaps in the literature by investigating the heterogeneous effects of parental divorce on family relationships, focusing on contact, closeness, and functional support. These effects are examined in relation to the pre-existing levels of these dimensions within families. Specifically, I investigate whether family relationships are less negatively affected by parental divorce in families with stronger solidarity and closer ties compared to those with weaker bonds. Consequently, the study focuses on whether changes in contact, closeness, and functional support are less pronounced in families that have higher levels of contact, closeness, and support prior to divorce.

I also explore whether gray parental divorce contributes to increased inequalities in family relationships, with families having weaker ties potentially being more severely impacted by divorce than those with stronger ties. Finally, given the differential impacts of gray parental divorce on fathers and mothers (Buyukkececi & Leopold 2024), the study investigates how these effects vary across mother–child and father–child relationships in families with varying levels of solidarity. For example, the negative consequences of parental divorce might be more severe for fathers than mothers in families with weak ties. As a result, I investigate the heterogeneous effects of gray parental divorce on both mother– and father–adult child relationships.

I use 13 waves of data from the German Family Panel to address these research questions. Germany offers several advantages as a study context, as it represents a relatively "typical" case within Europe. For example, in terms of intergenerational relationships, such as coresidence and contact levels, Germany holds a middle ground position between the "weaker" family ties seen in Nordic countries and the "stronger" ties typical of Mediterranean countries (Hank 2007). Moreover, trends in family dynamics, including the rise of gray divorce, in Germany align closely with those in other Western countries.

For the analyses, I use recentered influence functions (RIF) regression (Firpo et al. 2009), a method that goes beyond estimating average effects. This approach allows me to assess how parental divorce affects family relationships at different distribution levels of the outcome variables—specifically, contact, closeness, and functional support between parents and adult children. By examining changes in these dimensions of family solidarity following parental divorce, I determine whether the effects vary depending on the pre-divorce levels of contact, closeness, and support. Moreover, by comparing the consequences of parental divorce across different levels of family solidarity, this method enables to test whether gray parental divorce increases inequalities in solidarity among families.

## Background

### Intergenerational solidarity

Research on family solidarity has mainly built upon Bengston and Roberts’ (1991) multidimensional model to understand the nature and strength of intergenerational relationships (often referred to as solidarity). This framework, originating from social–psychological studies on small groups and families, encompasses six dimensions: (i) structural solidarity (such as geographic proximity), (ii) affective solidarity (representing emotional closeness or positive feelings), (iii) associational solidarity (measuring frequency of contact), (iv) functional solidarity (referring to both emotional and instrumental support), (v) consensual solidarity (assessing coherence in opinions, values, and perspectives), and (vi) normative solidarity (examining norms and expectations related to familistic values).

As one direct consequence of gray divorce is the experience of living alone without the support of a spouse, which can increase vulnerability and feelings of loneliness (Silverstein et al. 2006), this study focuses on the dimensions of solidarity that are most relevant to these experiences—namely, affective, associational, and functional solidarity. These dimensions are particularly relevant because they capture the core aspects of family relationships that gray divorcees typically rely on for emotional and practical support. Affective solidarity addresses emotional closeness and connection, associational solidarity relates to the frequency of contact and social interaction, and functional solidarity refers to the practical support provided within families (Bengtson & Roberts 1991). Moreover, these dimensions are the most extensively studied in the literature and are well-supported by long-term panel data (e.g., Buyukkececi & Leopold 2024).

### Gray parental divorce and parent–child relationships

The life course perspective emphasizes that individuals live in concert with relevant others whose behavior can influence relationships and life courses (Elder 1985). This framework extends to family relationships, which are crucial and subject to change, especially during significant life transitions (Buyukkececi et al. 2020; Buyukkececi & Leopold 2021; de Vuijst et al. 2017; Lyngstad & Prskawetz 2010).

Family systems theory builds on this by highlighting the interconnectedness of family dynamics, suggesting that life events and transitions can affect the entire family unit, not just individual members (Walsh 2016). These challenges can lead to either adaptation or maladaptation, with the latter referred to as vulnerability (McCubbin & Patterson 2014). A crisis may disrupt family functioning, either improving or worsening relationships (Patterson 2002).

In this context, much research has focused on the consequences of parental divorce for parent–child relationships, particularly in childhood (e.g., Aquilino 1994; Booth & Amato 1994; Rossi & Rossi 1990). These studies consistently show negative effects, regardless of children’s age. More recent work has explored “gray” divorce, occurring at or after age 50 (Buyukkececi & Leopold 2024; Lin et al. 2022). In the US context, Lin and colleagues (2022) found that divorced fathers reduced contact frequency but increased financial support to their adult children, while mothers doubled their interaction frequency without changing financial support patterns. Similarly, examining German families, Buyukkececi and Leopold (2024) documented strengthened mother–child solidarity but weakened father–child relationships post-divorce. These effects were most pronounced in contact frequency, followed by emotional closeness, and least evident in support exchanges.

While parental divorce generally has a negative effect on family solidarity, the extent of this impact varies across families. Family solidarity, defined as the strength and closeness of family ties, can buffer the disruptive effects of divorce, diminishing its negative impact on family bonds (McCubbin & McCubbin 1988, p. 247). Families with higher solidarity are better equipped to adapt to challenges through shared confidence, open communication, and collective problem-solving, helping them navigate crises like divorce (Black & Lobo 2008). These families often strengthen resilience through intra-family support, financial security, and reduced interparental conflict (Greeff & Van Der Merwe 2004). In contrast, families with lower solidarity may struggle to adapt, leading to more negative relationships (Jaffee et al. 2007; Tiet et al. 1998).

Families with higher levels of solidarity may be less likely to experience divorce, suggesting the potential for reverse causality. Stronger family ties, such as emotional closeness and frequent contact, may act as buffers against divorce, making the relationship between divorce and family dynamics more complex. This raises the need for caution when interpreting these associations. While acknowledging these considerations, divorce still occurs in families with varying levels of solidarity, and my analytical strategy allows for an examination of the consequences of gray parental divorce specifically for families with strong ties who have experienced divorce, moving beyond the average effects.

Building on existing literature (Buyukkececi & Leopold 2024; Lin et al. 2022), I hypothesize that *“gray” parental divorce is negatively related to parent–adult child relationships* (Hypothesis 1). However, *the severity of this relationship varies depending on the family’s level of solidarity* (Hypothesis 2). I expect families with lower solidarity to experience a more significant decline in parent–child relationships following divorce compared to families with higher solidarity. Consequently, *“gray” divorce increases inequalities in family relationships across different family types* (Hypothesis 3). Families with stronger solidarity may better navigate the challenges of divorce, while those with weaker solidarity may face more pronounced negative effects.

The gender of the parent could potentially moderate the impact of parental divorce on parent–child relationships (Leopold et al. 2024). Research has shown that father–child relationships are typically more vulnerable to the effects of divorce than mother–child relationships (de Graaf & Fokkema 2006; Kalmijn 2013). This is often attributed to fathers being less involved post-divorce, due to factors such as lower custody rates and a perception of fatherhood being linked to their role as a husband (Furstenberg & Cherlin 1991).

However, family solidarity may moderate this effect. In high-solidarity families, a supportive environment can help maintain strong relationships with both parents, even after divorce, buffering the negative impacts. Accordingly, I expect that *following “gray” parental divorce, disparities in the quality of mother–child and father–child relationships are more pronounced in families characterized by lower solidarity compared to families with higher solidarity* (Hypothesis 4). This is because divorce can further exacerbate these weaknesses or create new challenges among low-solidarity families. On the other hand, among families with high solidarity, the relationships with both parents might stay robust and even strengthen in the aftermath of divorce due to the supportive environment.

## Data

I used data from 13 waves of the German Family Panel (Pairfam; Brüderl et al. 2022), a longitudinal study that began in 2008. The initial sample included over 12,000 German residents from three birth cohorts (1971–1973, 1981–1983, and 1991–1993). In Wave 2, an additional 1,489 respondents from East Germany (DemoDiff) were incorporated, and Wave 11 introduced a new cohort (born 2001–2003) alongside a refresher sample of 6,000 respondents from the two younger cohorts. Although attrition peaked at 23% in Wave 2, it stabilized in subsequent waves, consistent with trends in other German surveys (Müller & Castiglioni 2015). Evidence further indicates that selective attrition is unlikely to have significantly affected the results (Müller & Schmiedeberg 2020).

The longitudinal design of Pairfam, spanning 13 years and capturing detailed information on parental marital status as well as intra- and intergenerational relationships, makes it exceptionally suitable to identifying families with varying levels of solidarity before parental divorce and analyzing how gray parental divorce affects family relationships compared to pre-divorce conditions.

To construct the analytic sample, I pooled all first-time respondents from both the initial wave and the refresher samples, totaling 18,912 individuals. I then restricted the sample to respondents aged 18 or older whose biological parents were both alive and married at the beginning of their observation period, resulting in 10,418 eligible individuals. Observations with missing data on father–child or mother–child relationships were excluded to ensure consistency across analyses. Over the course of the panel, 700 adult children experienced parental divorce.

Separate subsamples were created for each outcome measure in the analysis. While associational and affective solidarity were assessed in all waves, functional solidarity (emotional and instrumental support) was measured every other wave between Wave 2 and Wave 8. Details on the sample selection procedure and the total number of respondents and person-years for each outcome are provided in Figure A1 of Appendix.

**Fig. 1:**
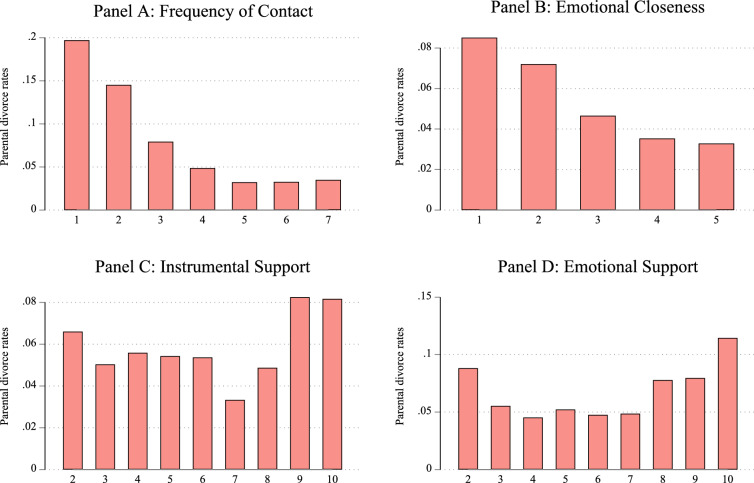
The Distribution of Gray Divorce Risk Across Parent–Child Relationship Dimensions. *Source*: Pairfam (Wave 1–13), release 13.0

### Measures

As the outcome measures of intergenerational solidarity, I focus on contact frequency (i.e., associative solidarity), emotional closeness (i.e., affective solidarity), and functional solidarity covering the instrumental and emotional support provided from children to their parents.

Associational solidarity was assessed with a single question asking respondents how frequently they maintained contact with their mother or father, including visits, letters, phone calls, and other modes of communication. The response scale ranged from 0 (never) to 6 (daily). Affective solidarity, on the other hand, was measured with a single item asking about the emotional closeness respondents felt toward their parents. The response options were categorized as: 1 (not close at all), 2 (somewhat less close than average), 3 (about average), 4 (somewhat closer than average), and 5 (very close). To measure instrumental support provided by children, the sum of two items was calculated: (i) frequency of providing help with shopping, household chores, or yard work and (ii) frequency of assisting parents in caring for other family members. Response categories ranged from 1 (never) to 5 (very often).

Emotional support from children was similarly assessed by combining the responses to two questions: (i) frequency of providing advice on personal problems and (ii) frequency of discussing parents’ worries and troubles. Response options were 1 (never) to 5 (very often). These composite measures were comparable to those employed in previous studies (Min et al. 2022). Additional analyses (available upon request) incorporating each individual item as an outcome separately yielded substantively consistent findings, supporting the reliability of the results despite the limited internal consistency. To ensure comparability across different outcomes, all four variables used in the main models were standardized.

The main predictor was a dummy variable, taking the value zero for all years preceding the divorce year and the value one for the divorce year and subsequent years. This approach allowed for an assessment of parent–adult child relationships and within-person changes both before and after parental divorce, as well as the identification of this association across different family solidarity levels. To account for age-related factors that might confound the association between parental divorce and intergenerational relationships, linear and quadratic age terms were included. This is because parent–child relationships may be influenced by normative age-related changes, such as children becoming more independent and intergenerational solidarity declining with age (Cooney 1994; Silverstein & Bengtson 1997). Age effects were controlled for using data from respondents who experienced parental divorce during the observation period as well as a control group comprising respondents who did not experience parental divorce. Descriptive statistics on variables of interest are shown in Table A1 in Appendix.

### Methods

To explore the association between parental divorce and intergenerational solidarity, I used recentered influence function (RIF) regression with individual-level fixed effects to analyze the unconditional quantiles of intergenerational solidarity on parental divorce, following Firpo et al.’s (2009) approach. Unlike conditional quantile regression, which conditions on the covariates, this strategy considers the entire distribution of the outcome variable. By considering the entire distribution, I can identify families with low, medium, and high levels of solidarity and assess the impact of parental divorce on each group separately. This approach allows to test for how different levels of family solidarity are affected by parental divorce, rather than just considering the average effect as in conventional fixed effects models.

By employing unconditional quantile regression models, I examined various points along the distribution of the outcome variable using the *rifhdreg* command introduced by Rios-Avila (2020), providing a more comprehensive understanding compared to traditional methods limited to average effects.

Furthermore, Firpo and colleagues (2009) employ this approach to calculate the unconditional partial effects (UPE) resulting from changes in the distribution of the independent variables on the distributional statistic of the outcome. Consistent with this, I employed RIF regression to further examine the unconditional partial effects (UPE) of parental divorce on inequality in intergenerational solidarity. While the Gini coefficient is a common measure of inequality, I calculated the variance as my outcome measure, as suggested by Rios-Avila (2020). This is because the RIF regression results for the Gini coefficient may be unreliable due to limitations in the influence function at the edges of the distribution. By analyzing the variance, I assessed whether parental divorce contributes to widening gaps in intergenerational solidarity across households as well as disparities father–child and mother–child relationships following parental divorce.

In all analyses, I combined respondent-reported outcomes for both mothers and fathers. For each individual, this approach yielded two observations per wave in the main analyses: one for the mother–child relationship and another for the father–child relationship. Consequently, the main results present combined estimated effects for mother–child and father–child relationships. Separate models for father–child and mother–child relationships are provided in Appendix as supplementary analyses.

To address disparities in father– and mother–adult child relationships following parental divorce, and to evaluate whether overall family solidarity moderates these differences, I further interacted the parental divorce dummy with the gender of the parent. This analysis showed how differences vary across different quantiles and distributions, specifically testing Hypothesis 3.

An important consideration, as previously discussed, is the potential for reverse causality, where parent–child relationships influence the likelihood of divorce rather than the other way around, which is the primary focus of this study. To address this issue and ensure the robustness of the findings, I conducted supplementary analyses using the Arellano-Bond generalized method of moments (GMM) estimator and an event study design combined with RIF regressions.

Moreover, to account for potential attrition bias, I employed a difference-in-differences (DID) approach with augmented inverse probability weighting (AIPW). This method diminishes bias from non-random dropout by reweighting observations based on their probability of remaining in the sample. The results of these robustness checks are presented in the *Supplemental Analyses* section.

## Results

Figure 1 illustrates the distribution of divorce rates across the four outcome measures analyzed in this study, based on respondents’ initial observations. The graphs show levels of family solidarity prior to parental divorce, even among respondents who experienced gray parental divorce. Panels A and B reveal that parent–adult child dyads characterized by lower contact frequency and emotional closeness were more likely to experience parental divorce later in life. Divorce rates steadily declined as contact frequency and emotional closeness between parents and adult children increased.

Similarly, with regard to instrumental and emotional support provided by children to their parents, divorce rates were higher among families where adult children provided low levels of support. However, this relationship followed a U-shaped pattern: Families with moderate levels of functional solidarity exhibited the lowest divorce risks, whereas those with both lower and higher levels of functional solidarity showed elevated risks, peaking among families with the highest levels of functional solidarity.

These findings, particularly those concerning contact frequency and emotional closeness, suggest that families with lower levels of solidarity are more vulnerable to parental divorce. If such families are less adaptable and less equipped to manage the challenges of divorce compared to high-solidarity families, parental divorce may exacerbate existing inequalities in intergenerational solidarity.

**Fig. 2:**
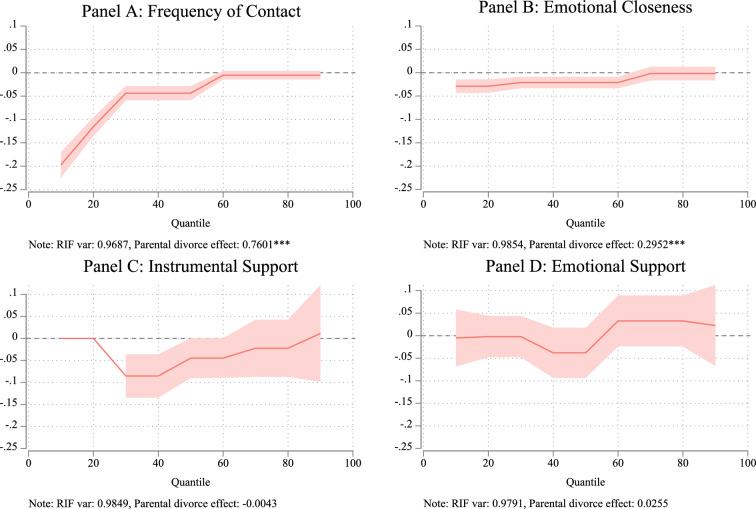
RIF Regression Results: Parental Divorce and Intergenerational Relationship Outcomes. *Source*: Pairfam (Wave 1–13), release 13.0. *Note*: Estimates of instrumental support provided by adult children to their parents were unavailable for the 10th and 20th quantiles due to an inadequate number of observations

Figure 2 illustrates the estimated effects of parental divorce on four measures of intergenerational solidarity, accounting for individual-level fixed effects and age (modeled quadratically). The analyses use pooled data on respondent-reported outcomes for both mothers and fathers, examining how parental divorce influences solidarity across different levels of the outcome variables. The findings suggest that gray parental divorce negatively affects parent–adult child relationships, supporting the first hypothesis. However, these effects vary depending on pre-divorce levels of family solidarity.

For contact frequency, families with low solidarity (10th quantile) experienced a reduction of approximately one-fifth of a standard deviation following parental divorce. This negative effect steadily diminished with increasing levels of solidarity, becoming statistically non-significant at the 60th quantile and above. This suggests that for high-solidarity families, parental divorce does not significantly impact contact frequency in later life.

A similar trend was observed for emotional closeness. Among low-solidarity families, emotional closeness decreased significantly after parental divorce. However, the adverse effects weakened for families with greater solidarity, becoming non-significant from the 70th quantile onward.

The negative effects of gray parental divorce on instrumental support were significant among low-solidarity families but diminished and became statistically non-significant beyond the median levels of solidarity. As shown in Panel D, parental divorce had no significant association with emotional support from children to parents across the distribution of the dependent variable. In high-solidarity families, the effects shifted from negative to positive, but these changes were also statistically non-significant.

Overall, the findings for contact frequency, emotional closeness, and instrumental support align with the second hypothesis, indicating that the adverse effects of parental divorce are diminished in families with high solidarity.

The bottom panels of each figure further show the average RIF variance and the marginal contribution of parental divorce to inequalities in intergenerational relationships. Analyses based on RIF regressions of variance for the four outcomes reveal that parental divorce significantly contributes to inequalities in contact frequency and emotional closeness but has no significant impact on inequalities in instrumental or emotional support provided by children to parents.

Specifically, the estimated effects indicate that if the proportion of gray parental divorce increased by 10 percentage points, the variance in contact frequency and emotional closeness would rise by 7.8% and 3.0%, respectively, calculated as (0.760/0.969 × 0.1) and (0.295/0.985 × 0.1). These findings provide partial support for the third hypothesis, suggesting that parental divorce in later life increases disparities in some aspects of intergenerational solidarity while leaving others unaffected.

### Gendered disparities in parent–child relationships

**Fig. 3:**
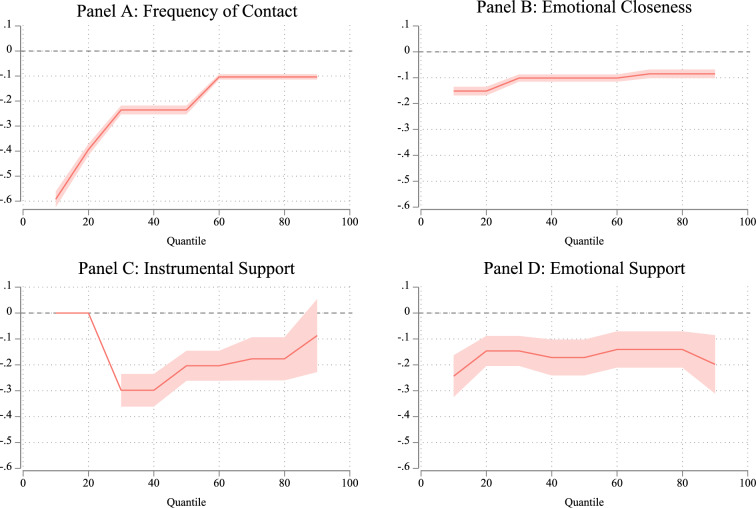
Impact of Parental Divorce on Mother–Child and Father–Child Relationships by Family Solidarity. *Source*: Pairfam (Wave 1–13), release 13.0. *Note*: Instrumental support estimates for adult children providing support to their parents were not available for the 10th and 20th quantiles

To examine differences between mother–child and father–child relationships following parental divorce, I analyzed the interaction between parental divorce and the gender of the parent. Figure 3 illustrates these interactions, highlighting how the effects of parental divorce vary between mothers and fathers and how these differences are changed by levels of family solidarity (full models are available from the author). Accordingly, the findings can be interpreted as how the gap in mother–child and father–child relationships changes following parental divorce across different levels of the outcome variable.

The observed patterns in these analyses align closely with the findings of Fig. 2. For contact frequency, emotional closeness, and instrumental support provided by children to their parents, the gap between mother–child and father–child relationships was significantly wider in families with low levels of solidarity following parental divorce. As family solidarity increased, this gap progressively narrowed. Importantly, the consequences of gray parental divorce for emotional support from children to their mothers and fathers did not show significant changes across the levels of family solidarity, as illustrated in Panel D.

Moreover, the parental gap remained consistent across all outcomes, indicating that the negative effects of parental divorce were more pronounced for fathers than for mothers, regardless of the level of family solidarity. In summary, these results support the fourth hypothesis: Differences between fathers and mothers in their relationships with children following gray parental divorce are more evident in low-solidarity families, while these disparities diminish in high-solidarity families.

### Supplementary analyses

To assess the robustness of the findings, I conducted several supplementary analyses. First, I used a difference-in-differences (DID) approach with augmented inverse probability weighting (AIPW) to account for attrition bias. This strategy addresses potential biases from non-random dropout by reweighting observations based on their probability of remaining in the sample. Moreover, this approach allows for the effects of parental divorce to vary over time with dynamic treatment effects, as shown in Appendix Figure A3. These dynamic effects allow to assess pre- and post-divorce effects and temporal changes. Accordingly, this also allows to test for reverse causality, as changes in parent–child relationships prior to divorce could suggest that relationship quality influences divorce likelihood rather than vice versa. Findings show that contact frequency, emotional closeness, and instrumental support provided to parents decrease significantly in the year of parental divorce, with these effects persisting for up to two years post-divorce. Overall, these results are consistent with the main findings (see, for instance, models focusing on medians in Table A2 in Appendix), suggesting that the associations in the main models are not driven by attrition bias.

Second, I used a dynamic panel model with the Arellano-Bond generalized method of moments (GMM) estimator to further address reverse causality. This model accounts for potential pre-existing dynamics in parent–child relationships that might influence the likelihood of divorce, by including lagged values of the dependent variable as covariates and using lagged values of parental divorce as instrumental variables. Findings shown in Table A3 of Appendix indicate that contact frequency, emotional closeness, and instrumental support decrease following divorce. These results provide additional evidence that the associations found in the main models are not driven by reverse causality.

Third, I replicated the RIF regression models using a conventional event study design. In this approach, two years prior to parental divorce serve as the baseline, with dummy variables capturing effects at five points: one year before, the year of divorce, one year after, two years after, and three or more years after divorce. These models test for anticipation effects while providing a more comprehensive picture of how the effects of parental divorce unfold over time and vary across families with different levels of solidarity. The results, shown in Figure A4 of Appendix, show no significant changes in parent–child relationships before divorce, further supporting the argument that reverse causality is unlikely. Importantly, models on contact frequency (Panel A) and instrumental solidarity (Panel B) show that post-divorce effects remain significant in the long-term among families characterized by lower solidarity. Accordingly, this highlights the heterogeneous effects of parental divorce, with families characterized by weaker ties experiencing longer-lasting and stronger effects in magnitude.

Lastly, I examined the robustness of the main findings by including additional time-varying factors that could influence the relationship between parental divorce and family solidarity (available from the author). This included travel time to parents, partnership and marital status, and the number of children. The results remained qualitatively robust to the consideration of these factors, supporting the reliability of the main findings.

## Conclusion

Close social relationships, particularly within families, are crucial for individual well-being, with the parent–child bond serving as one of the most influential ties (Fingerman et al. 2020). This relationship has been shown to buffer stress and promote positive outcomes for both parents and children (Bengtson 2001; Fingerman et al. 2020; Umberson et al. 2010).

Gray divorce may introduce stress into family dynamics. However, unlike spousal relationships, the parent–child bond remains stable (Cherlin 2010). As gray divorce rates rise and life expectancy increases, the importance of these relationships grows, especially with lower re-partnering rates (Silverstein et al. 2006). Maintaining strong intergenerational communication and support is crucial for promoting positive mental health outcomes in older adults (Fingerman et al. 2020). Strong intergenerational communication reduces loneliness and isolation, which are linked to poor mental and physical health in older adults (Blazer 2020). Theoretical perspectives suggest that families with higher solidarity are better equipped to cope with crises like parental divorce, while those with weaker social support may experience greater distress (Cohen & Hoberman 1983; Cohen & Wills 1985).

This study examines the relationship between gray parental divorce and intergenerational solidarity, focusing on whether stronger family ties mitigate the negative effects of divorce compared to weaker ties. I also investigate whether gray divorce exacerbates inequalities in family relationships, with weaker-tied families experiencing more severe impacts. Lastly, I examine how the effects of gray divorce differ between mother–child and father–child relationships and whether these differences vary across levels of solidarity.

Descriptive evidence shows that families with lower levels of solidarity, particularly in terms of contact frequency and emotional closeness, face a higher risk of gray divorce. Functional solidarity reveals a U-shaped pattern: The risk of divorce is elevated not only among families with low functional solidarity but also in those with high levels, potentially reflecting overinvolvement, which may increase tension and conflict.

Overall, my findings are consistent with previous literature on gray parental divorce and parent–adult child relationships (Buyukkececi & Leopold 2024; Lin et al. 2022). Observed trends indicated that gray parental divorce negatively impacts intergenerational relationships, with these effects being especially pronounced in father–adult child relationships. Recentered influence function (RIF) regressions show that the negative effects of divorce on parent–child relationships are most pronounced in low-solidarity families, which are also at higher risk of divorce. In contrast, families with medium to high levels of solidarity show a buffering effect, reducing the adverse impacts of divorce. Gender differences highlight that father–child relationships experience greater declines than mother–child relationships, especially in low-solidarity families. Although fathers receive significantly less emotional support than mothers following divorce, these differences remain consistent across varying levels of solidarity.

Understanding disparities in intergenerational solidarity is important for designing evidence-based policies that support vulnerable families. This is especially relevant in Western societies, where demographic trends such as increasing life expectancy, rising gray divorce rates (Buyukkececi & Leopold 2024; Lin et al. 2022), and a lower likelihood of re-partnering (Raley & Sweeney 2020) heighten the risk of loneliness and underscore the importance of parent–child relationships (Silverstein et al. 2006). This study highlights the need for targeted interventions aimed at strengthening family bonds and improving coping mechanisms, particularly for families with weaker ties (Umberson et al. 2016; Umberson & Thomeer 2020).

Promoting family integration and cohesion requires open communication, engaging in shared activities, and encouraging meaningful family rituals and traditions (e.g., Fiese et al. 2002). Programs that enhance open dialog and conflict resolution skills, community events and support groups that build and strengthen intergenerational relationships, and activities that uphold traditions across generations can significantly reinforce family bonds (Harrist et al. 2019).

Fathers navigating gray divorce often face unique challenges that necessitate tailored support (Buyukkececi & Leopold 2024). Providing culturally sensitive, gender-responsive counseling services, training professionals to address fatherhood-specific concerns, might be crucial to address gender disparities in parent–child relationships (Marsiglio & Pleck 2005; Pleck 2010).

While this study offers valuable insights, it also has limitations. First, the reliance on adult children’s perspectives highlights the need for complementary studies incorporating parental perspective for broader applicability. Second, although attrition rates stabilized in subsequent waves of the pairfam dataset (Müller & Castiglioni 2015), particular groups, such as low-solidarity families, might be underrepresented, although evidence using pairfam suggests that selective attrition is not likely to affect the results (Müller & Schmiedeberg 2020).

Another concern is the potential for reverse causality, where family relationships might influence the likelihood of divorce rather than being influenced by it. Although the findings were supported by robustness checks, including the Arellano-Bond generalized method of moments (GMM) estimator, an event study design combined with RIF regressions, and a difference-in-differences (DID) approach with augmented inverse probability weighting (AIPW), these methods may not fully eliminate the possibility of reverse causation. Consequently, the results should be interpreted with this consideration in mind.

Moreover, while findings were robust to the consideration of various time-varying factors, such as travel time between parents and adult children, partnership and marital statuses, and the number of children of the anchor, it is possible that other unobserved or dynamic factors—such as health status, or changes in caregiving needs—may also influence the association between parental divorce and parent–adult child relationships.

Future research could investigate how families with varying levels of solidarity respond to other significant life events, such as job loss, the death of a family member, marriage, or childbearing. These transitions, as highlighted by family systems theory (Minuchin 1985) and supported by recent findings (e.g., Buyukkececi & Çineli 2024), often reshape intergenerational dynamics. Understanding how these events influence both intra- and intergenerational relationships and the role of family solidarity in moderating these effects could offer valuable insights.

Moreover, the observed U-shaped pattern of functional solidarity invites further exploration into the consequences of excessive functional solidarity, from the perspectives of both parents and adult children. While research has examined the challenges of parental overinvolvement (Padilla-Walker et al. 2021), excessive involvement of adult children in their parents’ lives remains underexplored. Investigating this dynamic could enhance our understanding of intergenerational relationships and uncover the potential impact of such overinvolvement on the well-being of both generations.

## Supplementary Information

Below is the link to the electronic supplementary material.

Supplementary file1 (DOCX 609 KB)

## Data Availability

The German Family Panel (pairfam) data can be downloaded from GESIS after obtaining access: https://search.gesis.org/research_data/ZA5678.
