# Supplementary material for: Intergenerational relationships after parental divorce: variations by levels of family solidarity
Source: Eur J Ageing. 2025 Apr 30;22(1):19. doi: 10.1007/s10433-025-00849-x (PMC12043542; doi:10.1007/s10433-025-00849-x)
Supplement: Supplementary file 1 — Supplementary file1 (DOCX 609 KB) [file 10433_2025_849_MOESM1_ESM.docx]

**Appendix**

**Tables**

*Table A1 Descriptive Statistics*

|  | No Parental Gray Divorce | | Parental Gray Divorce | | Overall |
| --- | --- | --- | --- | --- | --- |
| Mother |  |  |  |  |  |
| Frequency of Contact | 6.12 |  | 5.87 |  | 6.10 |
|  | (1.12) |  | (1.49) |  | (1.15) |
| Emotional Closeness | 4.12 |  | 4.00 |  | 4.11 |
|  | (0.88) |  | (1.08) |  | (0.90) |
| Instrumental Support | 3.98 |  | 3.93 |  | 3.98 |
|  | (1.55) |  | (1.59) |  | (1.55) |
| Emotional Support | 5.46 |  | 5.71 |  | 5.48 |
|  | (1.75) |  | (1.97) |  | (1.77) |
| Father |  |  |  |  |  |
| Frequency of Contact | 5.92 |  | 4.85 |  | 5.84 |
|  | (1.26) |  | (1.90) |  | (1.35) |
| Emotional Closeness | 3.90 |  | 3.43 |  | 3.87 |
|  | (0.96) |  | (1.24) |  | (0.99) |
| Instrumental Support | 3.80 |  | 3.35 |  | 3.77 |
|  | (1.50) |  | (1.48) |  | (1.50) |
| Emotional Support | 4.74 |  | 4.52 |  | 4.73 |
|  | (1.68) |  | (1.90) |  | (1.70) |
| Sociodemographic Factors | | |  |  |  |
| Age | 29.42 |  | 26.29 |  | 29.19 |
|  | (9.29) |  | (8.64) |  | (9.28) |
| Personal Net Income | 1065.73 |  | 814.56 |  | 1046.83 |
|  | (1345.51) |  | (1015.41) |  | (1325.19) |
| Years of Schooling | 11.52 |  | 10.38 |  | 11.44 |
|  | (5.37) |  | (5.43) |  | (5.38) |

*Table A2 RIF Regression Results: Parental Divorce and Intergenerational Relationship Outcomes (standard errors in parentheses)*

|  | Contact frequency | Contact frequency | Contact frequency | Contact frequency | Emotional closeness | Emotional closeness | Emotional closeness | Emotional closeness |
| --- | --- | --- | --- | --- | --- | --- | --- | --- |
|  | Q(25) | Median | Q(75) | Variance | Q(25) | Median | Q(75) | Variance |
| Age | -0.024^***^ | -0.047^***^ | -0.068^***^ | -0.012^+^ | -0.000 | -0.003^**^ | -0.013^***^ | -0.016^**^ |
|  | (0.002) | (0.001) | (0.001) | (0.006) | (0.001) | (0.001) | (0.001) | (0.005) |
| Age-squared | 0.000^***^ | 0.001^***^ | 0.001^***^ | 0.000^***^ | -0.000 | -0.000 | 0.000^***^ | 0.000^**^ |
|  | (0.000) | (0.000) | (0.000) | (0.000) | (0.000) | (0.000) | (0.000) | (0.000) |
| Parental divorce | -0.115^***^ | -0.044^***^ | -0.005 | 0.760^***^ | -0.029^***^ | -0.021^***^ | -0.002 | 0.295^***^ |
|  | (0.010) | (0.008) | (0.005) | (0.041) | (0.007) | (0.006) | (0.007) | (0.034) |
| Constant | -0.047^+^ | 1.126^***^ | 2.130^***^ | 0.923^***^ | -0.768^***^ | 0.269^***^ | 1.416^***^ | 1.186^***^ |
|  | (0.024) | (0.018) | (0.011) | (0.097) | (0.017) | (0.015) | (0.017) | (0.079) |
| *N* | 106,658 | 106,658 | 106,658 | 106,658 | 106,296 | 106,296 | 106,296 | 106,296 |

|  | Instrumental support | Instrumental support | Instrumental support | Instrumental support | Emotional support | Emotional support | Emotional support | Emotional support |
| --- | --- | --- | --- | --- | --- | --- | --- | --- |
|  | Q(25) | Median | Q(75) | Variance | Q(25) | Median | Q(75) | Variance |
| Age | -0.052^***^ | -0.085^***^ | -0.132^***^ | -0.143^***^ | 0.006 | 0.014^**^ | 0.018^***^ | 0.003 |
|  | (0.005) | (0.004) | (0.006) | (0.013) | (0.004) | (0.005) | (0.005) | (0.010) |
| Age-squared | 0.001^***^ | 0.001^***^ | 0.002^***^ | 0.002^***^ | -0.000^+^ | -0.000^***^ | -0.000^***^ | -0.000 |
|  | (0.000) | (0.000) | (0.000) | (0.000) | (0.000) | (0.000) | (0.000) | (0.000) |
| Parental divorce | -0.085^***^ | -0.045^+^ | -0.022 | -0.004 | -0.002 | -0.038 | 0.033 | 0.025 |
|  | (0.025) | (0.023) | (0.033) | (0.070) | (0.023) | (0.028) | (0.029) | (0.058) |
| Constant | 0.456^***^ | 1.696^***^ | 3.149^***^ | 3.190^***^ | -0.595^***^ | -0.024 | 0.588^***^ | 1.059^***^ |
|  | (0.067) | (0.062) | (0.089) | (0.188) | (0.063) | (0.076) | (0.077) | (0.155) |
| *N* | 35,706 | 35,706 | 35,706 | 35,706 | 35,672 | 35,672 | 35,672 | 35,672 |

Source: Pairfam (Wave 1-13), Release 13.0, authors’ own calculations, ^+^ *p* < 0.10, ^*^ *p* < 0.05, ^**^ *p* < 0.01, ^***^ *p* < 0.001.

*Table A3 Arellano-Bond generalized method of moments (GMM) results*

|  | Contact frequency | Emotional closeness | Instrumental Support | Emotional Support |
| --- | --- | --- | --- | --- |
| Lagged dependent variable | 0.443 *** | 0.433 *** | 0.603 *** | 0.311 *** |
|  | (0.053) | (0.086) | (0.077) | (0.072) |
| Age | -0.088 *** | -0.014 *** | -0.061 *** | 0.004 |
|  | (0.008) | (0.003) | (0.012) | (0.007) |
| Age-squared | 0.001 *** | 0.001 * | 0.001 *** | -0.000 |
|  | (0.000) | (0.000) | (0.000) | (0.000) |
| Parental divorce | -0.374 *** | -0.118 * | -0.329 ** | -0.143 |
|  | (0.052) | (0.050) | (0.103) | (0.102) |
| Constant | 1.555 *** | 0.282 *** | 0.911 *** | -0.065 |
|  | (0.147) | (0.069) | (0.214) | (0.099) |
| Validity of instrument with Hansen test (Prob > chi2) | 0.000 | 0.000 | 0.200 | 0.714 |
| *N* | 83,788 | 83,436 | 22,078 | 22,110 |

Source: Pairfam (Wave 1-13), Release 13.0, authors’ own calculations, + p < 0.10, * p < 0.05, ** p < 0.01, *** p < 0.001.

**Figures**

*Figure A1 Sample selection*


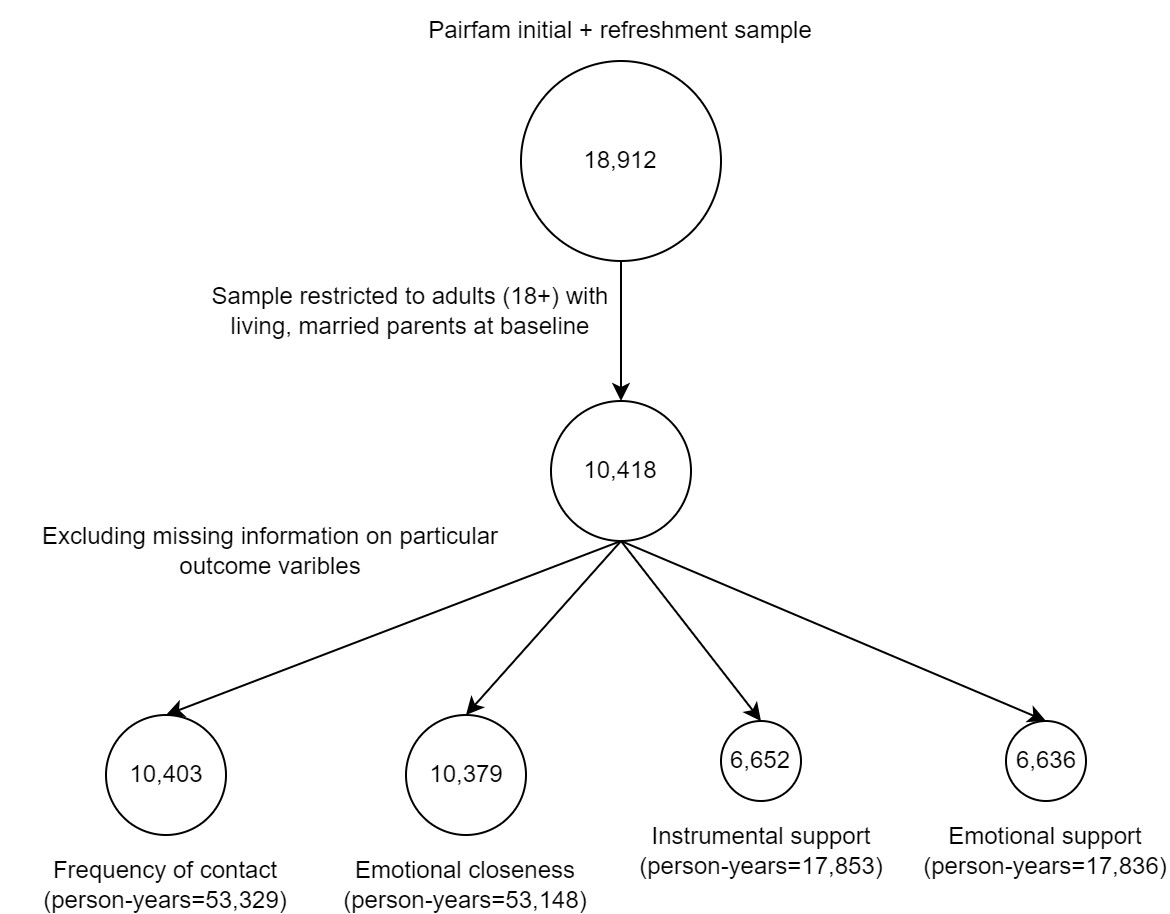


*Figure A3 Findings from RIF regressions separately for mothers and fathers*

**** Source: Pairfam (Wave 1-13), release 13.0.

Note: Instrumental support estimates for adult children providing support to their parents were not available for the 10th and 20th quantiles.

*Figure A4 Findings from* ***augmented inverse probability weighting (AIPW) models***

Source: Pairfam (Wave 1-13), release 13.0.

*Figure A5 Findings from RIF regressions with an Event Study Design*


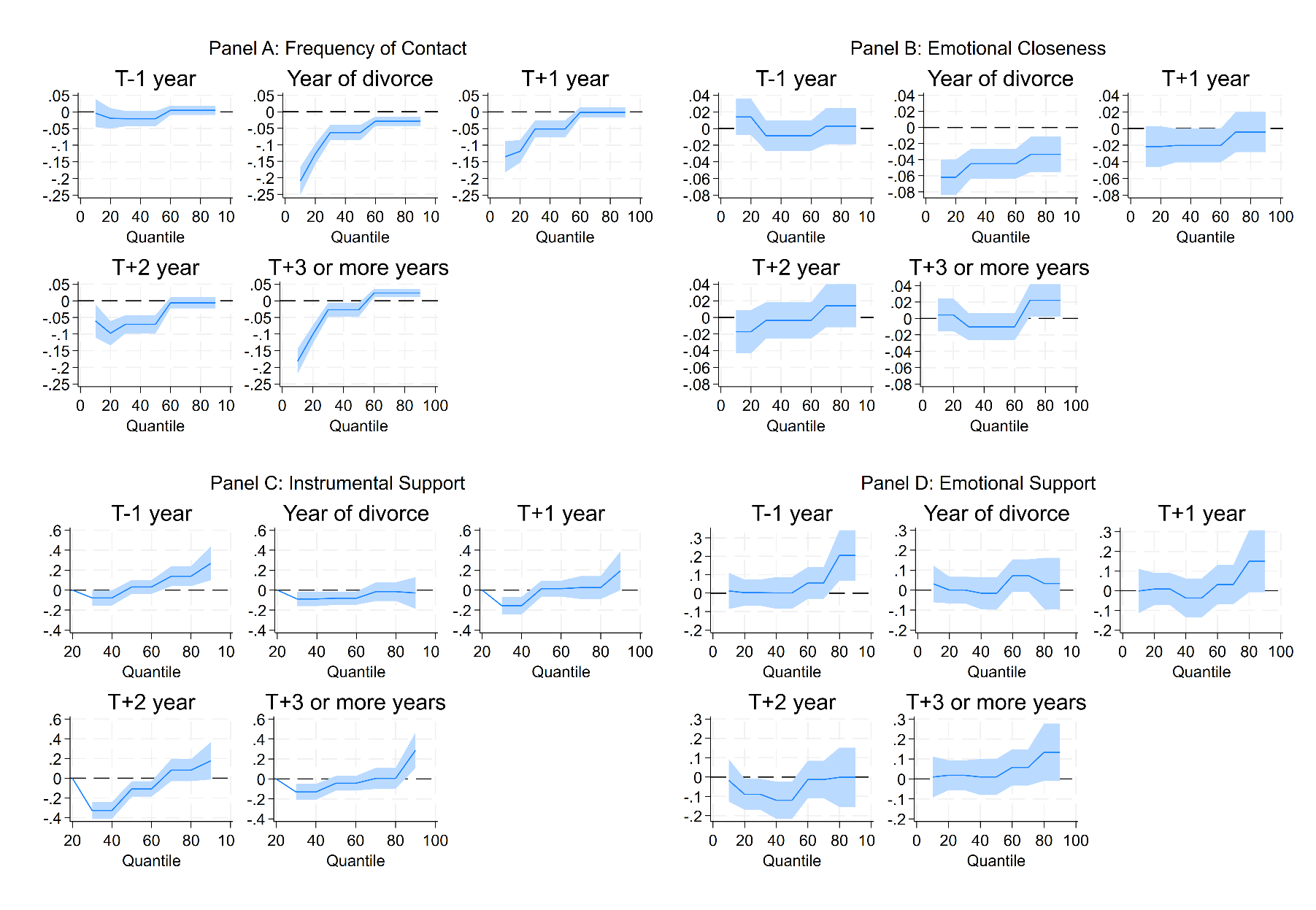


Source: Pairfam (Wave 1-13), release 13.0.
